# Quantitative Assessment of Murine Articular Cartilage and Bone Using X-Ray Phase-Contrast Imaging

**DOI:** 10.1371/journal.pone.0111939

**Published:** 2014-11-04

**Authors:** Jun Li, Huihui Yuan, Mingshu Wu, Linan Dong, Lu Zhang, Hongli Shi, Shuqian Luo

**Affiliations:** 1 School of Biomedical Engineering, Capital Medical University, Beijing, China; 2 Department of Rheumatology and Immunology, Capital Medical University, Beijing, China; University of Pittsburgh, United States of America

## Abstract

Murine models for rheumatoid arthritis (RA) research can provide important insights for understanding RA pathogenesis and evaluating the efficacy of novel treatments. However, simultaneously imaging both murine articular cartilage and subchondral bone using conventional techniques is challenging because of low spatial resolution and poor soft tissue contrast. X-ray phase-contrast imaging (XPCI) is a new technique that offers high spatial resolution for the visualisation of cartilage and skeletal tissues. The purpose of this study was to utilise XPCI to observe articular cartilage and subchondral bone in a collagen-induced arthritis (CIA) murine model and quantitatively assess changes in the joint microstructure. XPCI was performed on the two treatment groups (the control group and CIA group, n = 9 per group) to monitor the progression of damage to the femur from the knee joint in a longitudinal study (at 0, 4 and 8 weeks after primary injection). For quantitative assessment, morphologic parameters were measured in three-dimensional (3D) images using appropriate image analysis software. Our results showed that the average femoral cartilage volume, surface area and thickness were significantly decreased (P<0.05) in the CIA group compared to the control group. Meanwhile, these decreases were accompanied by obvious destruction of the surface of subchondral bone and a loss of trabecular bone in the CIA group. This study confirms that XPCI technology has the ability to qualitatively and quantitatively evaluate microstructural changes in mouse joints. This technique has the potential to become a routine analysis method for accurately monitoring joint damage and comprehensively assessing treatment efficacy.

## Introduction

Rheumatoid arthritis (RA) is characterised by progressive synovial joint inflammation, which results in cartilage erosion and bone destruction [1], [2]. Due to the damage to the joints, RA patients suffer from chronic pain, deformity of the joints, and physical disability [3]. Although analysis based on semi-quantitative scoring has contributed to an understanding of RA, detailed structural information on joint damage during RA progression is lacking. To provide sufficient information for the diagnosis and treatment of RA, accurate assessments of the structural changes to joints are needed. Small animal models, which are necessary and complementarity to clinical studies, are well suited for understanding pathogenesis and developing drugs. The collagen-induced arthritis (CIA) mouse model can reliably mimic clinical RA and is an ideal model for morphologic research on joint damage [4].

The current techniques for evaluating joint morphologic changes, such as histologic scoring and biochemical assays, are time consuming, require specimen destruction and cannot provide 3D structural information [5], [6]. Magnetic resonance imaging (MRI) and ultrasonography (US), which are non-destructive 3D imaging techniques, are commonly used for the examination of rheumatoid joints in the clinic [7]–[9]. Unfortunately, these two imaging methods are not feasible to monitor the articular cartilage injury in CIA mouse. At present, the normal spatial resolution of MRI is in the range of 3–5 mm and a few can reach up to 1 mm [10]. High-quality US used for musculoskeletal system even are capable of scanning at spatial resolution of 100 µm [11]. However, compared with the thickness of the cartilage in mice joints, they still are not able to provide adequate spatial resolution for the measurement of thinner joint cartilage in mice. Because the articular cartilage of a normal mouse has an average thickness of 50 µm [12]. So, due to the limitation of spatial resolution, the small lesions in mouse joints are difficult to be detected by MRI and US. In addition, for US, the most important limitation pertains to penetration, that makes it is unable to detect much beyond the bone interface and into the deeper articular structure [10]. In contrast, micro-computed tomography (micro-CT) can provide superb high-resolution at the level of several microns for visualising alterations to the micro-architecture of bone tissue in mice [4], [14]. However, biological soft tissues such as articular cartilage are generally undetectable by micro-CT due to low X-ray attenuation [15]. Therefore, the imaging methods described above all have limitations for the visualisation of mouse joints.

X-ray phase-contrast imaging (XPCI) is a novel imaging technique that combines the advantages of high spatial resolution, high phase contrast and 3D non-invasive imaging. It greatly improves the image quality of biological soft tissues. It is well known that X-ray is an electromagnetic radiation. When it propagates through a sample, which may be absorbed, scattered or traverse the sample without interaction. These variations result in changes of the transmitted intensity and the phase shift. This can be described by the complex refractive index n = 1-δ-iβ. The imaginary part β describes the absorption image which is mainly attribute to the X-ray attenuation. Because the variation in X-ray absorption between different biological soft tissues is very small. So, this may lead to low contrast and poor resolution. The real part δ is ascribed to Thorson scattering which is related to the X-ray phase shift. The latter is three orders of magnitude higher than the imaginary part for biological soft tissues. Therefore, the information of X-ray phase shift can be used to achieve an image contrast in sample which do not exhibit significant absorption. Meanwhile, it can provide quite high spatial resolution, even less than 1 µm. Therefore, XPCI is quite suitable for imaging the fine structure to biological soft tissues.

XPCI as a unique method has proven to be a powerful technique for analysing the composition and structure of biological tissues [16], [17] and materials [18], [19]. To date, this method has been successfully utilised to visualise human articular cartilage [20] and detect damage to mouse articular cartilage [21], [22]. Furthermore, a few researchers have applied this technique to assess and quantify changes in the articular cartilage of mice [12]. However, these studies have mostly focused on the destruction of articular cartilage rather than subchondral bone. In reality, these two tissues are intimately related. Anatomically, they are physically connected to one another. Functionally, they play synergistic roles in the conduction of stress [23]. The morphologic changes in one of these tissues inevitably affect the structure and function of the other [24], [25]. Thus, simultaneously assessing the destruction of cartilage and bone is crucial for investigating the mechanisms of joint diseases.

The aim of this study was to apply the XPCI technique to the simultaneous visualisation and assessment of articular cartilage erosion and bone loss in a mouse RA model. First, XPCI was used to image the femur of the knee joint in normal and CIA mice. Then, 3D morphologic images of the femoral cartilage, subchondral bone surface and trabecular bone were reconstructed to qualitatively describe the structural changes. In addition, to further enhance the reliability and objectiveness of the results, the morphologic parameters of the articular cartilage and trabecular bone were measured in the 3D images to provide a quantitative assessment.

## Materials and Methods

### Samples

8-week-old male DBA/1 mice (CIA-susceptible mouse strain, H-2q) were purchased from Beijing HFK Bio-technology Co., Ltd (Beijing, China). All mice were maintained under specific pathogen free conditions and fed standard rodent chow ad libitum. All experimental procedures conformed to the Chinese National Health and Capital Medical University Guidelines and were approved by the Institutional Animal Ethics Committee of the Beijing Military Medical Institution (Permit Number SYXK2007-005).

18 mice were randomly assigned to the two treatment groups: 9 mice for the control group and 9 mice for the RA group. RA was induced by a subcutaneous injection with 100 µL of an emulsion containing 100 µg of bovine type II collagen (CII, Sigma-Aldrich, St Louis, MO) and 100 µg of complete Freund's adjuvant (Sigma-Aldrich, St Louis, MO) at the tail. After 21 days, 100 µl of a booster (100 µg of bovine CII in incomplete Freund's adjuvant) was injected subcutaneously at the tail, but at a different location from the site of first injection. The mice were monitored daily for feeding and signs of pain throughout the experimental period. The mice were killed by cervical vertebra dislocation at 0 week (at the beginning of experimentation) as well as at 4 and 8 weeks after primary immunisation. The fresh femora of the knee joints (left and right) were dissected from the surrounding tissues and immediately fixed in phosphate-buffered formaldehyde (4%, PH 7.0) at 4°C for 24 h.

### X-ray phase-contrast imaging

**Figure 1:**
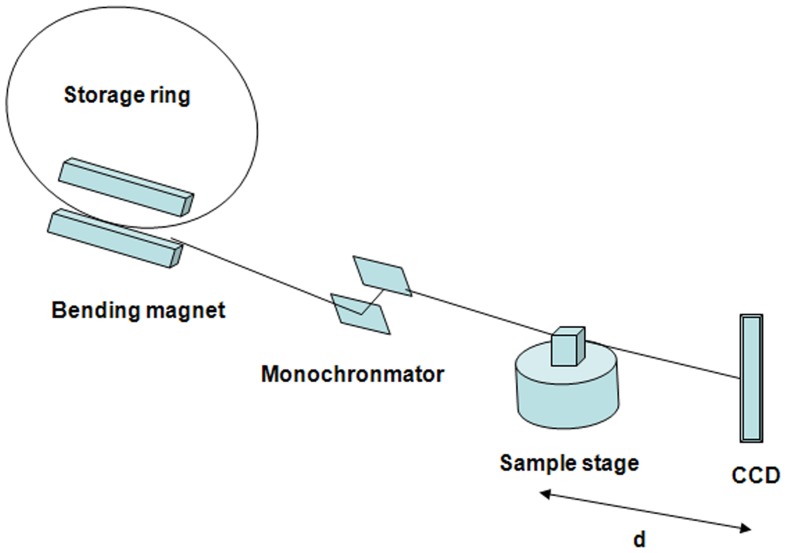
Schematic of the experimental setup of the XPCI system. Femur samples were positioned vertically on the sample stage. During the CT imaging, the sample stage rotated 180° automatically. A CCD camera was used to record the imaging results. In our experiment, the distance between the sample stage and CCD detector was set to 15 cm.

The experiment was conducted at beamline BL13W1 of the Shanghai Synchrotron Radiation Facility (SSRF) in China. A diagram of the experimental setup is shown in Figure 1. The X-ray synchrotron radiation beam line was emitted from a storage ring of electrons with an acceleration voltage of 3.5 GeV and an average beam current of 180 mAn. Subsequently, the beam was monochromatised by a Si(111) double-crystal. The energy range was 8–72 keV, with an energy resolution of ΔE/E<5e-5. After the monochromatic beam penetrated through the sample, it was detected by a thin(100 µm) CdWO_4_ cleaved single-crystal scintillator. The projections were recorded by an X-ray sensitive CCD camera. The distance between the sample stage and the CCD camera could be adjusted from 0 to 3 m by moving the CCD.

Because XPCI can provide the quite high contrast for visualization of articular cartilage. In this work, we did not use contrast reagents. Based on our previous experiment, XPCI was carried out using an energy of 14 keV. The distance between sample and the CCD camera was 15 cm. The CCD camera had a resolution of 1768×1768 pixels for a 3.7 µm×3.7 µm area. Seven hundred and twenty projections were recorded (the sample stage was rotated in steps of 180°/720), with an exposure time of 2 seconds for each projection. For the analysis, a volume of interest (VOI) was selected for each sample that extended 4 mm down from the top surface of the cartilage and included cartilage, subchondral bone lamella, and some trabecular bone.

### Histological Processing

After the final XPCI scan, the femora of the knee joints were decalcified in 20% EDTA at 4°C for 6 weeks. Subsequently, the specimens were dehydrated using a series of gradient alcohol treatments (immersion in 70%, 80%, 90% alcohol for 3 hours and in 100% alcohol for 4 hours). After dehydration, the samples were embedded in paraffin. For comparison with the 2D images generated by XPCI, the paraffin-embedded samples were sectioned at a thickness of approximately 4 µm. Finally, the sections were stained with Safranin-O using standard protocols.

### Image processing

Several factors can influence the quality of projections in the imaging process, such as background noise and ring artifacts. Thus, image preprocessing is crucial prior to analysis. First, We applied the method of image normalisation to remove noise signals from the background. The projection removed background can be written as
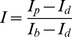
(1)Where, I_p_ is the projection acquired from XPCI. I_b_ is the background image and I_d_ is dark field image.

Then, persistent ring artifacts were eliminated by the method of sinogram correction. Actually, the ring artifacts in the reconstructed CT image were due to the vertical stripe artifacts in the sinogram. Therefore, in this method, all operations were performed on the sinogram image instead of the projecton. The first step was to recognise the position of the vertical stripe in the sinogram. And then, a local median filter was executed at this position to depress the vertical stripe. The details of this method were briefly outlined below:

1. Perform the filtering in the sinogram P(β,s). The filtered sinogram P_h_(β,s) can be described as:
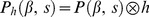
(2)

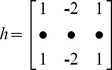
(3)where, h was the filter operator whose size was able to be adjusted according to the noise level. β was the angle of projection, and s was the coordinates of projection. The aim of this step was to enhance the signal of vertical stripe in the sinogram in order to detect the stripe effectively.
2. Calculate the average 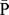
(s) of each row of the filtered sinogram Ph(β,s).
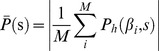
(4)Where, M was the total number of projections. After the average, the signal intensity at the position of vertical strip was stronger than others. It would form a peak signal, which was the foundation for the subsequent recognition.
3. Calculate the maximal value 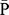
_max_ of 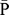
(s). When 0<c≤1, if
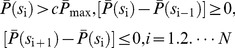
(5)Coordinates i was detected and regarded as the position of the vertical stripe. Here N was the width of the probe. c was an experimentally determined threshold. Through adjusting the value of c, the ring artifacts of different strength was able to be recognised.
4. After detecting the position of all vertical stripes, the correction was done by using the median filter in the positions of the detected stripes only. By using the above method, we gained the corrected sinogram.

**Figure 2:**
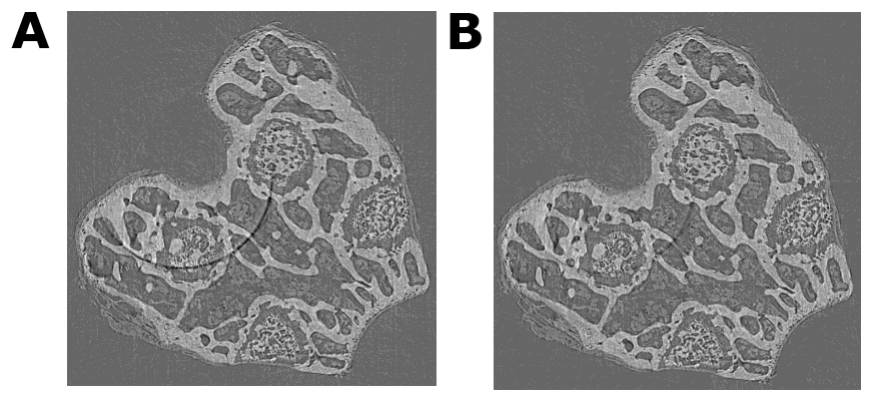
Before and after CT image via image preprocessing. (A) 2D CT image without image preprocessing. (B) 2D CT image after image preprocessing.

After the above steps, the conventional filtered back projection (FBP) algorithm with a Hamming filter was used to reconstruct CT slices from the 720 projections. The femora were placed in a vertical position during scanning. Therefore, a series of transverse cross-sectional slices was produced. Before and after CT image of removing ring artifacts are shown in Figure 2. We found that the ring artifacts were suppressed effectively, and the quality of image had improved significantly.

**Figure 3:**
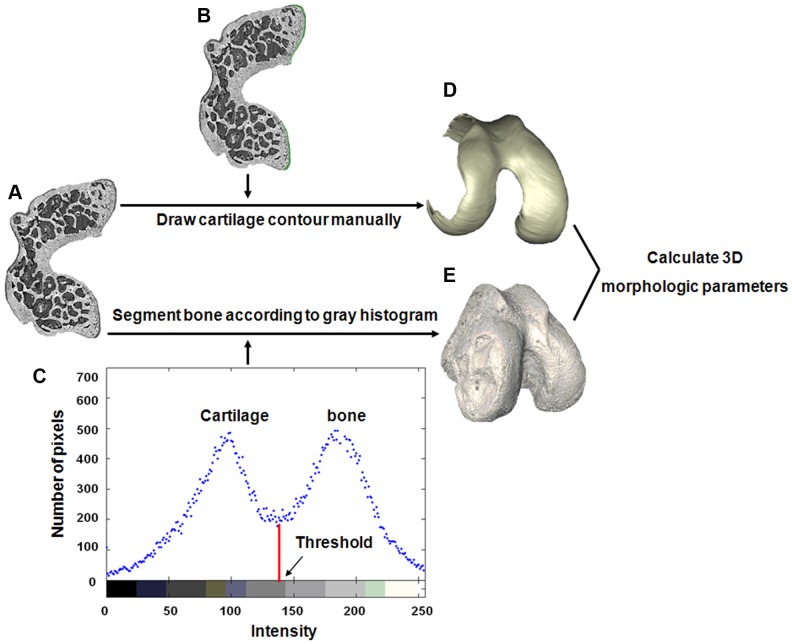
Segmentation methods for quantifying cartilage and bone morphology in the mouse femur. 2D XPCI image of a femoral axial section (A). The method of region growing and manual drawing was used to semi-automatically segment the cartilage, which is indicated in green (B). According to the grayscale histogram (C), an appropriate threshold, indicated by the arrowhead, was selected to separate the bone from the femur. Through the use of the surface rendering method, the 3D morphologies of the femoral cartilage (D) and bone (E) were visualised to calculate the 3D morphologic parameters.

Femoral cartilage and subchondral bone are adjacent in the anatomic structure. Thus, for objective analysis of the morphologic changes in these two tissues, it was necessary to separate them from the femur. We applied an image segmentation method based on region growing to separate the cartilage, which was a semi-automatic procedure. It started in manually choosing the seed point in the region of cartilage as well as setting the intensity threshold. Under the control of the intensity threshold, the growing would stop when the cartilage were separated. However, on account of the morphologic disconnection of cartilage, this semi-automatic segmentation approach was not feasible for all the CT slices. To overcome this limitation, the CT slices unsuitable for the above method were loaded in Photoshop software (Adobe, California, USA), and by drawing contour lines to manually extract the femoral cartilage (this part of the work was performed under the guidance of two animal experiment experts). For segmentation of the bone, an appropriate threshold was easy to select. According to a grayscale histogram analysis of the tissues, the valley value in the midpoint of two partially overlapping peaks could be considered as an optimal threshold to separate the bone from the whole femur automatically. After the image segmentation, the 3D morphologies of the entire articular cartilage and subchondral bone were visualised using a surface rendering method. The segmentation methods for the femoral cartilage and bone are shown in Figure 3.

In addition, for quantitative analysis, the 3D data sets of the femoral cartilage and trabecular bone were analysed in Amira software, version 5.2.2 (FEI, Portland, USA) and CTAn software, version 1.8.1.2 (SkyScan, Kontich, Belgium), respectively, to measure the following morphologic parameters: cartilage volume (mm^3^), cartilage surface area(mm^2^), cartilage average thickness (µm), bone volume to tissue volume ratio (BV/TV, %), bone surface to bone volume ratio (BS/BV, mm^−1^), trabecular bone thickness (Tb.Th, mm), trabecular bone number (Tb.N, /mm) and trabecular bone space (Tb.Sp, mm).

### Statistical analysis

The 3D morphologic parameters of the femoral cartilage and trabecular bone at different time points (0, 4 and 8 weeks after primary injection) were compared using a one-factor repeated generalised linear model with Tukey's test analysis. The data are presented as the mean±standard deviation (SD). P values less than 0.05 were considered statistically significant. Statistical analysis was performed with SPSS, version 17.0(SPSS Inc, Chicago, IL).

## Results

### Histological analysis

**Figure 4:**
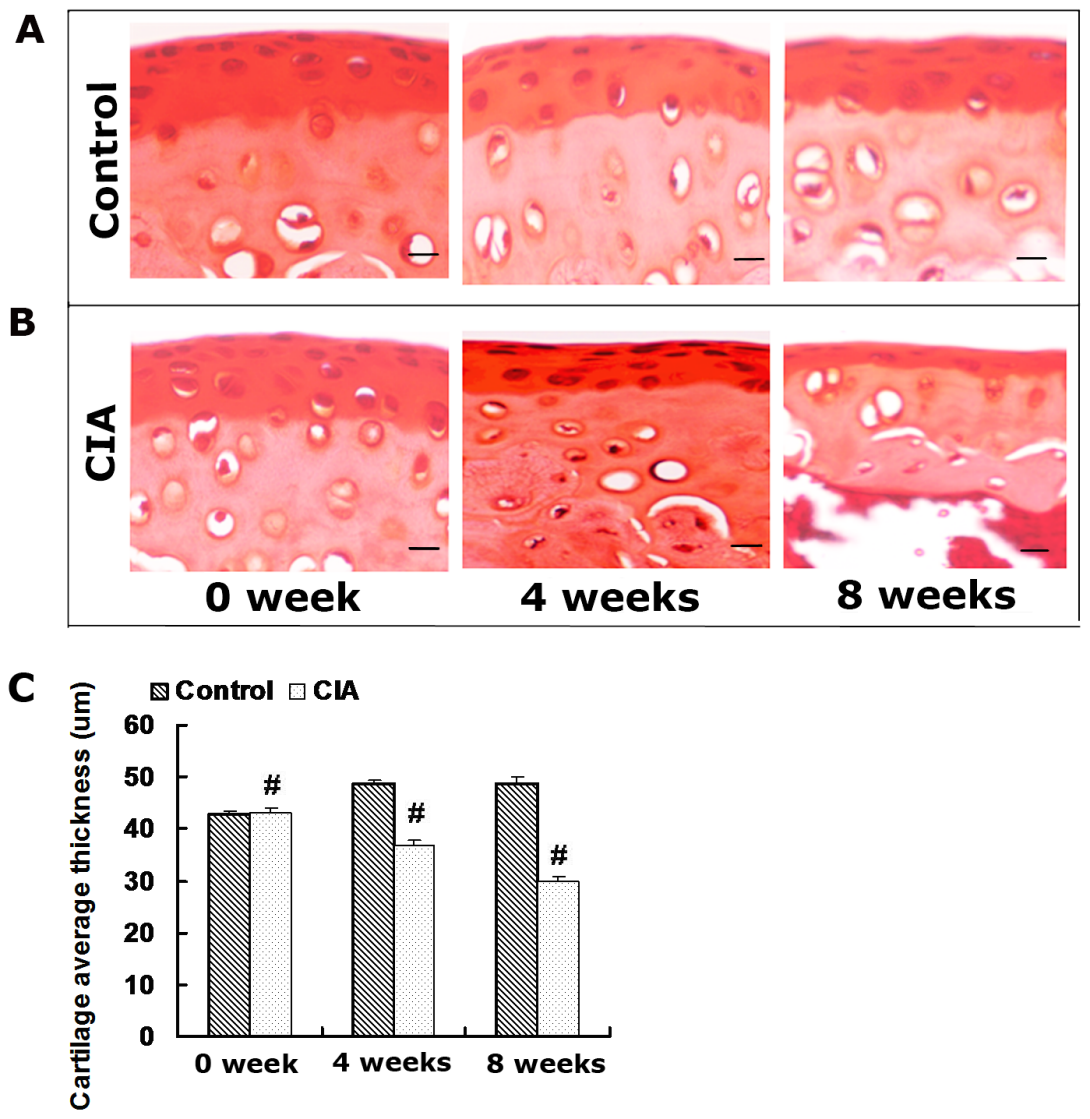
Histological images of femur sections stained with Safranin-O. (A) Femur sections from control group at 0, 4 and 8 weeks after the primary injection. (B) Femur sections from CIA group at 0, 4 and 8 weeks after the primary injection. (C) Average thickness of femoral cartilage assessed by histology. Data are presented as the the mean±SD. 2D CT image before image preprocessing. 2D CT image before image preprocessing. #: P<0.05 for differences between the control group and the CIA group. Scale bar: 20 µm.

Representative images of femur sections stained with Safranin-O and the from control and CIA group are shown in Figure 4. At 0 weeks after primary injection, the contour line of the femoral cartilage layer was relatively smooth and the subchondral bone was basically intact. In contrast, at 4 and 8 weeks after primary injection, the thickness of the femoral cartilage was decreased significantly and the contour line was not smooth. Furthermore, part of the subchondral bone surface was obviously eroded. By statistic analysis, the average thickness of femoral cartilage form CIA group at 4 and 8 weeks were respectively low 21% and 34% compared to control group (P<0.05).

### Morphologic analysis by XPCI

**Figure 5:**
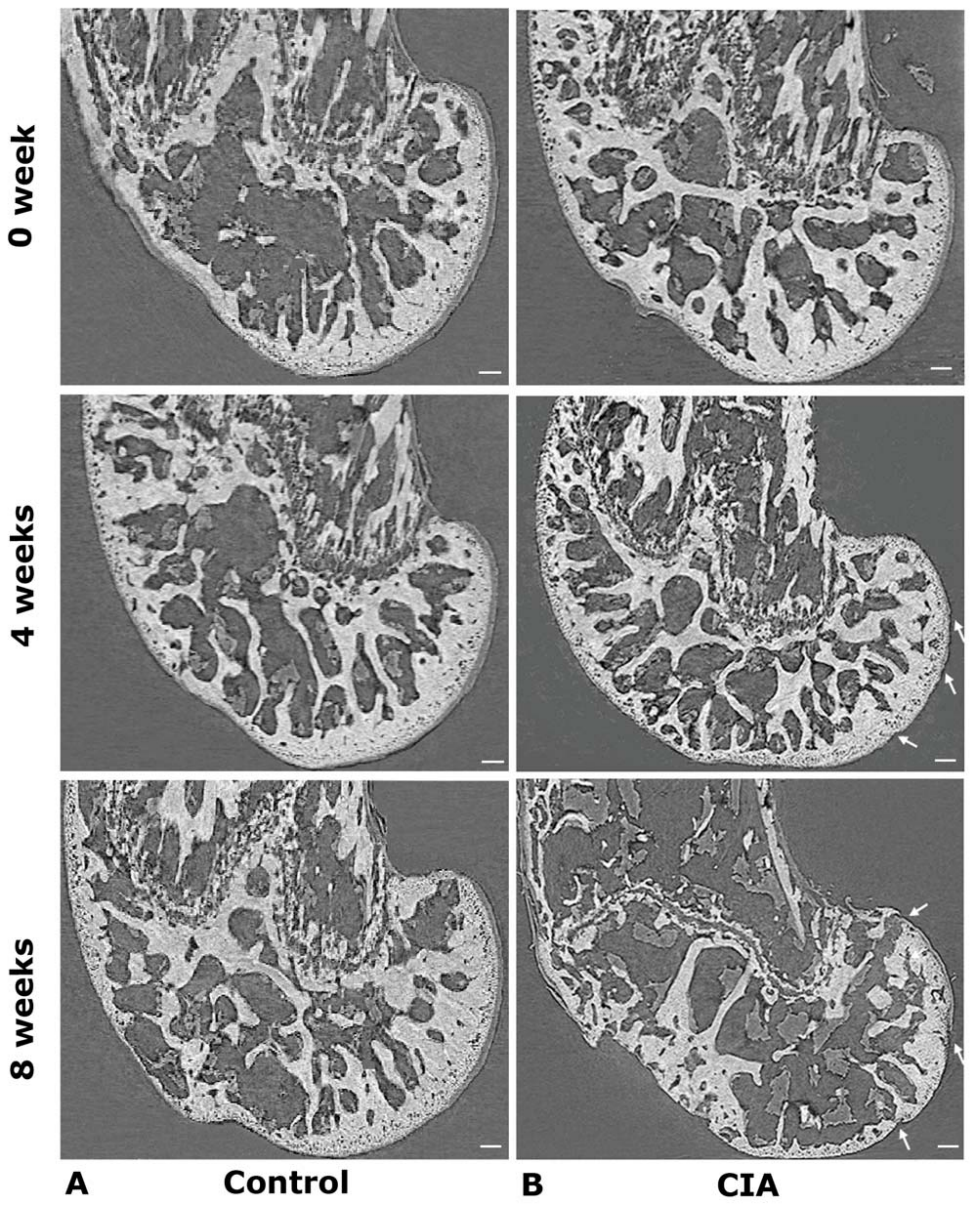
2D XPCI sagittal images of femora. (A) Femur from a normal mouse at 0, 4 and 8 weeks after the primary injection. (B) Femur from a CIA mouse at 0, 4 and 8 weeks after the primary injection. The red arrowheads indicate the damaged regions in the femora. Scale bars: 100 µm.

Representative 2D XPCI images are shown in Figure 5. The imaging results clearly show structural changes in the femoral cartilage and subchondral bone in the CIA mice with the RA progression. At 0 weeks after the primary injection, the surface of the subchondral bone was covered with smooth and intact cartilage, and the thickness of the cartilage was uniform. There were no obvious difference between the control and CIA group. At 4 weeks after the primary injection, a portion of the cartilage and bone surface were destroyed, and the cartilage thickness was also decreased significantly compared with the control group. At 8 weeks after the primary injection, femoral structure of normal mouse did not have any significantly change. But, in the CIA group, most of the cartilage was destroyed and had fallen off. Meanwhile, subchondral bone loss was extensive, with more than 50% of the surface having been eroded. These imaging results derived from XPCI correlated well with the histological analysis.

**Figure 6:**
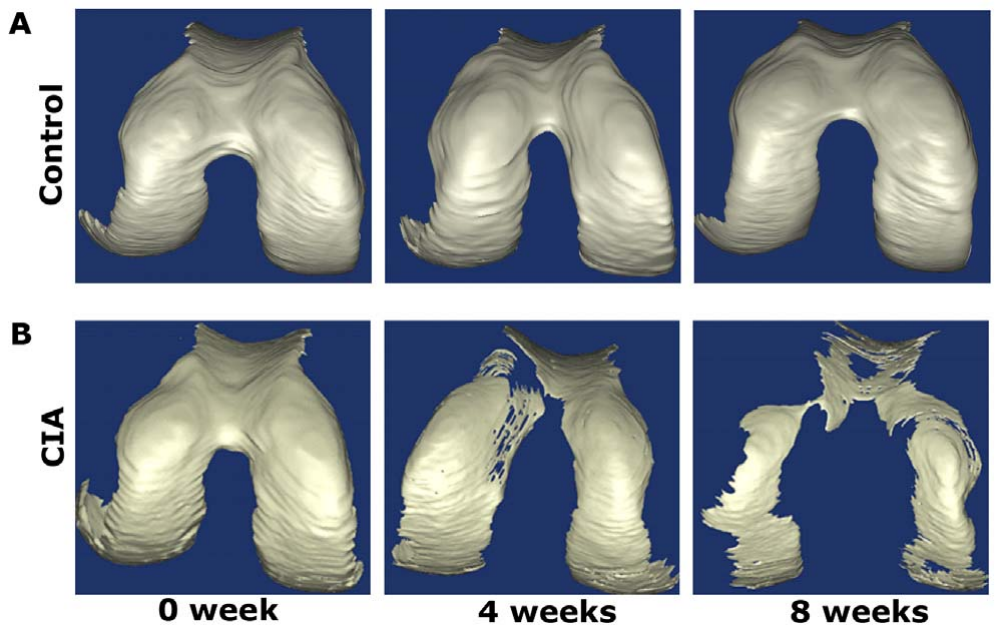
The 3D morphologic images of femoral cartilage. (A) Femoral cartilage from a mouse in control group at 0, 4 and 8 weeks after the primary injection. (B) Femoral cartilage from a mouse in CIA group at 0, 4 and 8 weeks after the primary injection.

**Figure 7:**
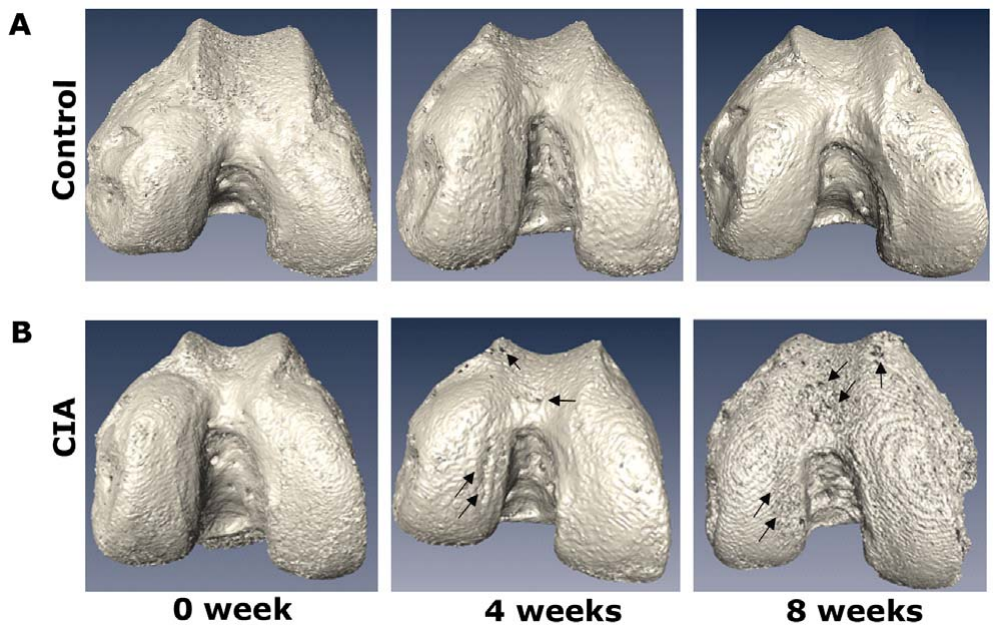
The 3D morphologic images of subchondral bone sufaces. (A) subchondral bone from a mouse in control group at 0, 4 and 8 weeks after the primary injection. (B) subchondral bone from a mouse in CIA group at 0, 4 and 8 weeks after the primary injection. The black arrowheads indicate the damaged regions of the subchondral bone surface.

**Figure 8:**
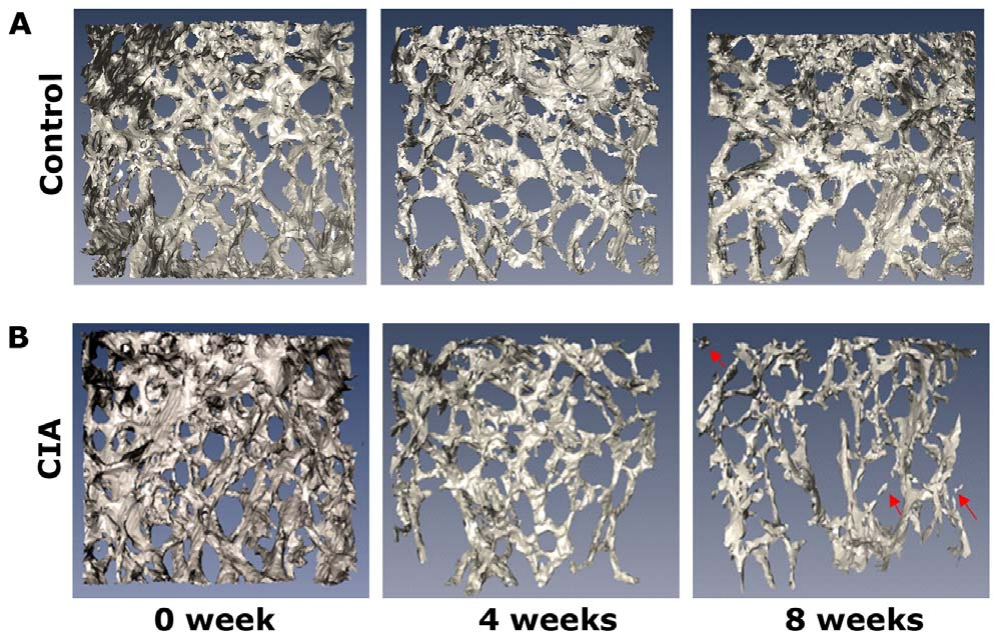
The 3D morphologic images of Trabecular bone. (A) Trabecular bone from a mouse in control group at 0, 4 and 8 weeks after the primary injection. (B) Trabecular bone from a mouse in CIA group at 0, 4 and 8 weeks after the primary injection. The red arrowheads indicate fragments of trabecular bone.

The surface rendering method was used to visualise the femur, which provided an intuitive means of identifying 3D architectural anomalies in the femoral cartilage, subchondral bone surface and trabecular bone. Representative 3D images are respectively shown in Figure 6, 7 and 8. At 0 weeks after the primary injection, the structure of femur was basically intact, and no cartilage erosion, subchondral bone lamella denudation or trabecular bone deformities were apparent. As time progressed, we observed that the outer edge of the femoral cartilage, where synovium is densely distributed, was eroded relatively earlier. At later time points, the cartilage damage gradually expanded from the outside to the inside and had an aggravating tendency with the development of the disease. Meanwhile, the subchondral bone was also invaded. Part of the subchondral bone surface was destroyed at 4 weeks, particularly in regions without cartilage coverage, and it had further deteriorated at 8 weeks. At 4 weeks, obvious architectural destruction of the trabecular bone was observed in the form of decreased trabecular bone number and increased trabecular bone separation at 4 weeks. At 8 weeks, there was a progressive increase in the number of bone fragments compared to 4 weeks.

**Figure 9:**
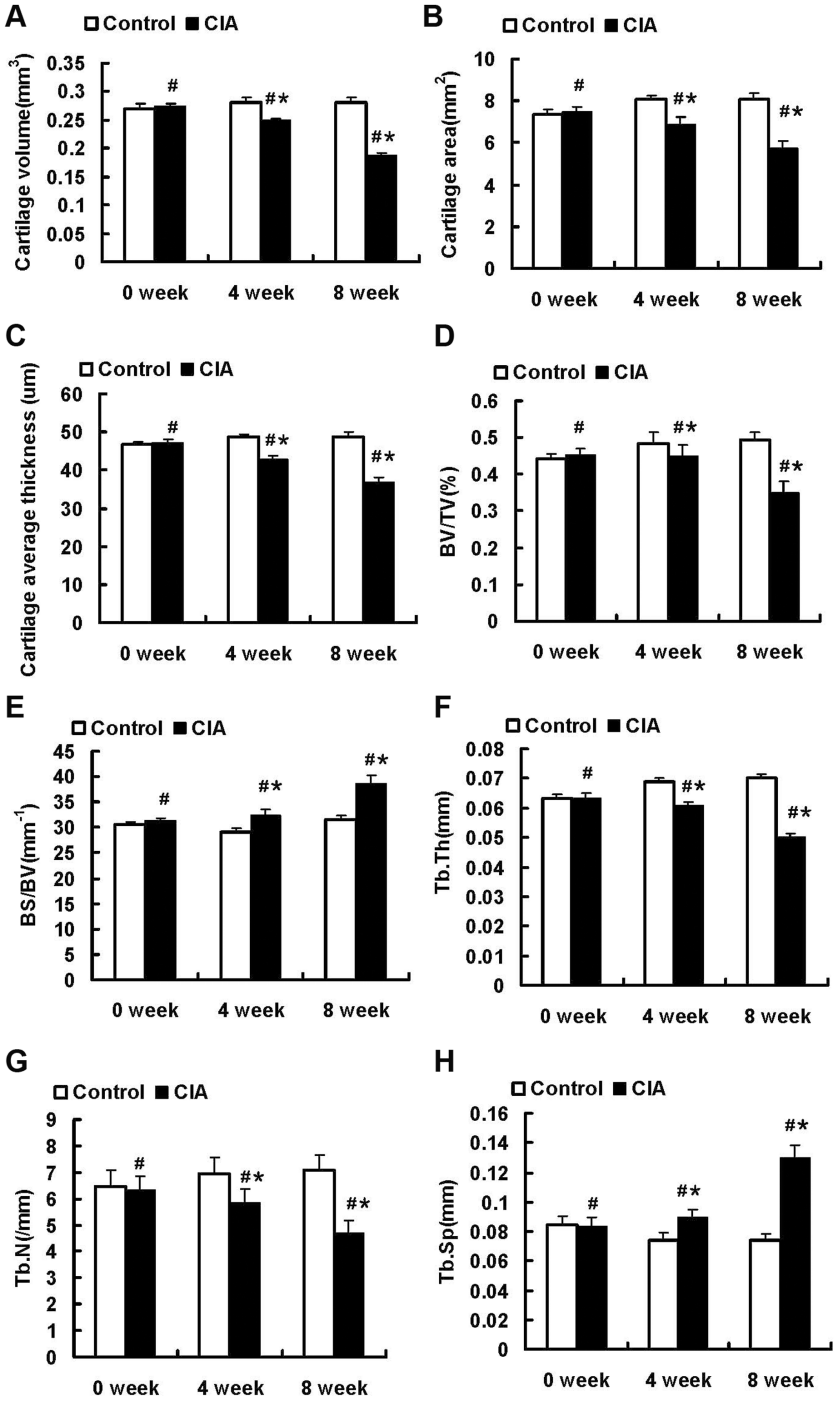
Evaluation of femoral morphology changes between control and CIA groups. (A) Cartilage volume. (B) Cartilage surface area. (C) Average cartilage thickness. (D) Bone volume to tissue volume ratio (BV/TV). (E) Bone surface to bone volume ratio (BS/BV). (F) Trabecular bone thickness (Tb.Th). (G) Trabecular bone number (Tb.N). (H) Trabecular bone space (Tb.Sp). Data are presented as the mean±SD. *: P<0.05 for differences between the control group and the CIA group; #: P<0.05 for differences within the CIA group.

To objectively analyse the structural changes in the articular cartilage and subchondral bone in a quantitative manner, the femoral 3D morphologic parameters of two treatment groups (the control group and CIA group) were measured (Figure 9). At 4 weeks after the primary injection, the femoral cartilage volume, surface area and average thickness in the CIA group were 11%, 15% and 12% lower, respectively, than in the control group (P<0.05). In addition, BV/TV, Tb.Th and Tb.N in the CIA group were 6%, 11% and 14% lower, respectively, than in the control group (P<0.05), whereas BS/BV and Th.Sp were 10% and 16% higher, respectively (P<0.05). At 8 weeks after the primary injection, the cartilage volume, surface area and average thickness in the CIA group were decreased by 32%, 28% and 26%, respectively, compared to the control group (P<0.05). Moreover, BV/TV, Tb.Th and Tb.N in the CIA groups were decreased by 28%, 29% and 34%, respectively, corresponding increases of 18% and 43% were measured for BS/BV and Tb.Sp, respectively, compared to the control group (P<0.05).

Within the CIA group, the 3D morphologic structure obviously changed in the femora over time. From 0 to 4 weeks, the articular cartilage volume, surface area and average thickness decreased by 7%, 6% and 10% (P<0.05), respectively, BV/TV, Tb.Th and Tb.N decreased by 7%, 10% and 9% (P<0.05), respectively, and BS/BV and Th.Sp increased by 6% and 7% (P<0.05), respectively. At 8 weeks, the femoral cartilage and bone were further eroded compared to the 0 week time point. The cartilage volume, surface area and average thickness were decreased by 20%, 17% and 14%, respectively, and these decreases were accompanied by reductions in BV/TV, Tb.Th and Tb.N by 22%,17% and 26%, respectively (P<0.05). Meanwhile, BS/BV and Th.Sp were increased by 15% and 35%, respectively (P<0.05).

## Discussion

Although 2D histological analysis is currently regarded as the accepted standard for evaluating the loss of cartilage and bone [6], 3D images obtained from XPCI can provide more structural information for integrated morphologic analysis and precise volumetric assessment. Moreover, histological processing may produce artifacts caused by sample dehydration, embedding and sectionaing in the measurement of morphologic parameters [26]. In contrast, XPCI is a non-invasive imaging method that not only allows for repeated measurements of the same sample but also simplifies the preparative work before imaging to eliminate the possible artifacts and improve the work efficiency.

High-resolution MRI enables 3D non-destructive imaging of cartilage tissue at a resolution of approximately 50 µm [27]. Some studies have used this method to measure the articular cartilage thickness in healthy rats [28] and OA guinea pigs [29]. However, even for high-resolution MRI, according to this level of resolution, quantitative imaging for the accurate measurement of cartilage thickness and volume in a RA mouse is still limited, because the knee joint cartilage of a RA mouse has an average thickness of 30–60 µm. Moreover, the intrinsically poor contrast between cartilage and joint fluid makes it difficult to distinguish them by MRI [13], which may make it difficult to interpret pathological changes.

Compared with routine micro-CT, micro-CT arthrography improves the deficiency of X-ray insensitivity to soft tissues, making it possible to observe morphologic changes in the articular cartilage of mice [26]. However, this technique relies on the use of X-ray absorbing contrast agents that could lead to a risk of comorbidity, such as joint infection or allergic reaction. In addition, another limitation is that micro-CT arthrography, at best, provides functional rather than truly structural information because of differential binding of contrast agents to healthy versus diseased cartilage [21].

Importantly, RA is a condition that affects both articular cartilage and subchondral bone. Elucidating the pathology of these two tissues is important for the comprehensive diagnosis and treatment of RA. The optimal simultaneous imaging of cartilage and bone is not possible with current MRI and CT systems. In contrast, XPCI can provide high resolution, an order of magnitude higher than MRI, and distinguish the border between cartilage and other tissues without the need for contrast agents [30]. This technique presents opportunities to realise repetitive and accurate measurements of cartilage and bone tissues. Thereby, we adopted XPCI to study progressive joint damage in a mouse RA model.

The CII induced murine RA model has excellent repeatability, sensitivity and reliability. Furthermore, it specifically demonstrates progression of human RA in a timely manner [31]. The use of CII as an exogenous alloantigen stimulates an autoimmune response and induces pannus formation [32]. The articular cartilage is a barrier covering the surface of the subchondral bone, and it is attacked first by destructive enzymes and inflammatory mediators. Following damage of the articular cartilage, the exposed subchondral bone is eroded. When the structure of the articular cartilage is badly destroyed, the pressure and friction on the subchondral bone are increased [33], [34]. This interplay between biochemical and mechanical factors causes a vicious cycle of cartilage damage and bone loss. In our study, the measurements made by XPCI definitively showed morphologic changes in the joint, including obvious decreases in cartilage thickness, area, volume, BV/TV, Tb.Th and Tb.N, as well as corresponding increases in BS/BV and Tb.Sp.

## Conclusions

XPCI technology has ability to qualitatively and quantitatively evaluate changes in the joints of RA mice. Moreover, the analysis of 3D morphologic parameters by XPCI here revealed important pathologic features during RA progression. This technique can potentially provide new insights to improve the understanding of RA pathogenesis and be extended to the development of new therapeutic strategies. We believe that with instrumentation and methodological developments, this advanced imaging method has the potential to be applied broadly for the comprehensive and accurate evaluation of joint diseases.
